# FM0807 decelerates experimental arthritis progression by inhibiting inflammatory responses and joint destruction via modulating NF-κB and MAPK pathways

**DOI:** 10.1042/BSR20182263

**Published:** 2019-09-03

**Authors:** Nanwen Zhang, Zhiwei Liu, Hongbin Luo, Weifang Wu, Kaimei Nie, Lin Cai, Shuangyu Tan, Xiaole Chen, Ying Huang, Jiaxing Liu, Meina Lv, Xin Zhang, Yun Fan, Yuying Lin, Shennan Ye, Yang Liu, Lixian Wu, Jianhua Xu

**Affiliations:** 1 School of Pharmacy, Fujian Medical University, Fuzhou, P.R. China; 2 Key Laboratory of Natural Medicine Pharmacology in Fujian Province, Fuzhou, P.R. China; 3 School of Clinical Medicine, Fujian Medical University, Fuzhou, P.R. China; 4 The First Affiliated Hospital of Fujian Medical University, Fuzhou, P.R. China; 5 Department of anesthesia, Fuzhou Children’s Hospital of Fujian Province, Fuzhou, P.R. China

**Keywords:** inflammation, MAPK, NF-κB, rat, rheumatoid arthritis

## Abstract

Background: Rheumatoid arthritis (RA) is a chronic articular synovial inflammatory disease. The precise etiology underlying the pathogenesis of RA remains unknown. We aimed to investigate the inhibitory effect of curcumin analog FM0807 (curcumin salicylate monoester, 2-hydroxy-, 4-[(1E,6E)-7-(4-hydroxy-3-methoxyphenyl)-3,5-dioxo-1,6-heptadien-1-yl]-2-methoxyphenyl ester) on experimental RA and investigate its possible mechanisms of action.

Method: Rats with Freund’s complete adjuvant (FCA)-induced arthritis (AIA) were administered aspirin (0.1 mmol.kg^−1^), curcumin (0.1 mmol.kg^−1^), FM0807 (0.1, 0.2 mmol.kg^−1^) and vehicle via gastric gavage, from days 7 to 21, once daily. The hind paw volume and arthritis index (AI) were measured, and radiographic and histological examinations were performed. Twenty-one days later, the animals were killed and left ankle joints were removed to measure protein expression of the elements of the nuclear factor κB (NF-κB) and mitogen-activated protein kinase (MAPK) pathway by Western blot analysis. The enzyme-linked immunosorbent assay (ELISA) was employed to measure synovial fluid levels of tumor necrosis factor-α (TNF-α), interleukin (IL)-6, IL-1β and IL-10.

Results: Compared with AIA group, FM0807 reduced the AI and swelling of the injected hind paw in a dose-dependent manner, and inhibited increases in inflammatory cell infiltration, pannus formation and cartilage destruction. FM0807 also potently attenuated the increase in the expression of inflammatory factors TNF-α, IL-6 and IL-1β in synovial fluid, while IL-10 levels were also elevated. FM0807 significantly suppressed phosphorylation of extracellular-signal-regulated kinase (ERK) 1/2 (ERK1/2), c-Jun-N-terminal kinase (JNK) 1/2 (JNK1/2), p38MAPK, inhibitor of NF-κB kinase (IKK), IκB and NF-κB p65 protein, (all *P*<0.05), which displayed more potential effects compared with those of the aspirin and curcumin groups.

Conclusion: FM0807 exerts its therapeutic effects on RA by inhibiting cartilage degeneration. FM0807 treatment might be an effective therapeutic approach for RA.

## Introduction

Rheumatoid arthritis (RA) is a chronic articular synovial inflammatory disease, the main features of which are articular cartilage and bone erosion caused by synovial joint system inflammation. RA causes significant morbidity, mortality and significant socioeconomic burden. It has an estimated population prevalence of 0.5–1%, with a higher female preponderance, and is more common in the northern than in the southern hemisphere [1]. The precise etiology underlying the pathogenesis of RA is unknown; however, it is thought to be triggered by genetic and environmental factors [2]. A large body of evidence has outlined the broad molecular and physiological mechanisms of the inflammatory reflexes that modulate pro-inflammatory cytokines, including tumor necrosis factor-α (TNF-α), interleukin (IL) 6 (IL-6) and IL-1β and macrophage switching. Furthermore, clinical data support the theory that RA may decrease levels of the anti-inflammatory cytokine IL-10. Several lines of evidence suggest that the mitogen-activated protein kinase (MAPK) families, p38, c-Jun-N-terminal kinase (JNK) and extracellular-signal-regulated kinase (ERK) regulate expression of these inflammatory cytokines and play vital roles in RA pathogenesis [3].

The prevention and treatment of RA have been a topic of much interest over the years. However the underlying mechanisms of RA are complex, and yet the therapeutic treatment of RA still requires improvements [4]. Because of the severe complications of RA and the side effects associated with conventional drug therapy, the quality of life and compliance of patients with treatment are severely affected. For example, non-steroidal anti-inflammatory drugs (NSAIDs) and cyclooxygenase-2 (COX-2) inhibitors relieve symptomatic pain; however, they cannot prevent the disease from progressing. They are also associated with adverse gastrointestinal and cardiovascular effects. The use of biologics (antibodies or soluble receptors for TNF-α, IL-1β and IL-6) also increases the risk of opportunistic infection, including fungal or mycobacterial infections, which can present with nonspecific symptoms such as appetite loss, low-grade fever, cough, shortness of breath and skin lesions or other systemic symptoms [5–7]. In spite of the fact that biologic agents have been recently accepted clinically, treatment with disease-modifying anti-rheumatic drugs (DMARDs), are often preferred because of low costs and the less risk of side effects such as hypersensitivity, infections or oncogenesis. Among the DMARDs, methotrexate (MTX) is generally accepted as the first-line drug for RA treatment [8]. However, MTX has an extensive toxicity range, which is the main cause of therapy withdrawal [9]. MTX treatment is discontinued in 8–19% of patients due to adverse reactions that include gastrointestinal, hepatic, renal, pulmonary and hematological disturbances, and may also affect the central nervous system. Patients often are forced to change the dose or switch to a different drug [10]. Therefore, the search for effective and less-toxic drugs for the treatment of RA continues [11].

**Figure 1:**
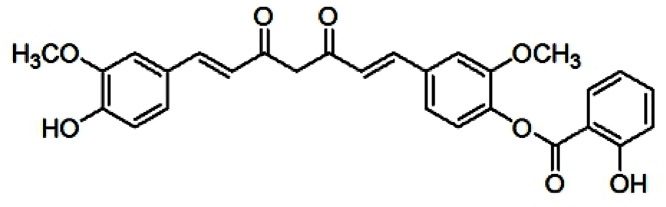
Chemical structure of FM0807 used in the experiment The exact mass of FM0807 is 488.15.

Curcumin has attracted great attention due to its anti-neoplastic and anti-inflammatory activities, such that it is widely used as a treatment for various diseases. The molecular mechanisms of curcumin involve not only the suppression of cell proliferation and metastasis, but also the down-regulation of various factors, including nuclear factor κB (NF-κB), IL-1β, TNF-α and activator protein, all of which are associated with the pathogenesis of RA [12]. Through several clinical trials, curcumin has been proved to be safe and effective [13]. RA patients, who received curcumin (1200 mg/day) for 2 weeks suffered no discernible side effects [14]. Nevertheless, its poor water solubility and low bioavailability restrict its use. To address this problem, a number of derivatives and analogs of curcumin have been developed in our laboratory, including FM0807 (curcumin salicylate monoester, 2-hydroxy-,4-[(1E,6E)-7-(4-hydroxy-3-methoxyphenyl)-3,5-dioxo-1,6-heptadien-1-yl]-2-methoxyphenyl ester)) that incorporates a salicylate into curcumin and retains the β-dike tone structure (Figure 1); it exhibits better anti-inflammatory activity than curcumin [15]. Previously, we demonstrated that FM0807 was very stable in high humidity environments, and its degradation was undetectable until the tenth day under high temperature. The oil/water partition coefficient of FM0807 fell in the interval of 1–2 in buffer solutions with various pH values. The half-life of FM0807 in various solutions of pH 2–9 was longer than 2 days. FM0807 is more stable than its precursor: curcumin [16]. It was shown that FM0807 had a substantial inhibitory effect on acute and chronic inflammation [17,18]. We hypothesized that the drug could also exert a therapeutic effect on RA.

Considering that inflammation is a core feature of RA, we speculated that FM0807 may also be effective in RA. Here, we tested the effects of the drug in an adjuvant-induced arthritis (AIA) rat model. Specifically, we measured inflammatory factors, ankle morphology and protein expression of the MAPK pathway. We hope to make a pharmacodynamic evaluation regarding the effect of FM0807 on RA and to provide a theoretical basis for the development of new medications. The effects of FM0807 on RA and its possible mechanisms will be demonstrated in the present study for the first time.

## Materials and methods

### Animals

Forty-eight sterile male Sprague–Dawley (SD) rats, weighing 160 ± 20 g, were provided by the Experimental Animal Center of Fujian Medical University (Certificate No. SCXK (Fujian) 2016-0002). All experimental animals were raised in non-toxic plastic containers, with each cage containing four rats. Cages were cleaned every 1–2 days. Ambient temperature was 20–25 degrees and humidity was 60–70%. The animals were housed with food and water being provided *ad libitum*. After 1 week of acclimatization to laboratory conditions, all rats were weighed and randomly assigned. The experimental animals were maintained under standard laboratory conditions at room temperature with 12-h light/dark cycle. All procedures were performed in accordance with protocols approved by the Ethics Review Committee for Animal Experimentation of Fujian Medical University (No. 2017-047). All animals were raised in Laboratory Animal Center of Fujian Medical University and animal handling procedures were performed in strict accordance with the care of laboratory animals according to the Fujian Province Zoological Society.

### Groups and model establishment

Eight rats were selected randomly from the 48 (SPF grade) as the normal control group. Each rat was intradermally injected with saline at 0.1 ml in the left hind paw pads, while the other 40 rats were injected with an equal volume of Freund’s complete adjuvant (FCA) in the left hind paw pads to establish the AIA rat model according to the method of Rice et al. [19]. This was designated as day 0. The model animals were randomly assigned to five groups (*n*=8 in each group) on day 7: Vehicle control group, Curcumin group (AIA + 0.1 mmol.kg^−1^ per day intragastric administration of curcumin), Aspirin group (AIA + 0.1 mmol.kg^−1^ per day intragastric administration of aspirin), FM0807-I group (AIA + 0.1 mmol.kg^−1^ per day intragastric administration of FM0807) and FM0807-II group (AIA + 0.2 mmol.kg^−1^ per day intragastric administration of FM0807). Simultaneously, the normal and the vehicle groups were intragastrically treated with saline daily. Clinical evaluation was performed prior to immunization (baseline) and on alternate days following the initiation of drug treatment (post-dosing) up to 21 days, including standardized arthritis scores and measurements of edema [20].

### Drugs and reagents

Complete Freund’s adjuvant (Chondrex in the United States, 10 mg.ml^−1^, lot: 140522), aspirin (purity = 99%) and Cur (purity = 99%) were purchased from the Third Reagent Factory of Shanghai, China, curcumin analog FM0807 (curcumin salicylate monoester, 2-hydroxy-, 4-[(1E,6E)-7-(4-hydroxy-3-methoxyphenyl)-3,5-dioxo-1,6-heptadien-1-yl]-2-methoxyphenyl ester) was provided by New Drugs Institute of Fujian Medical Univerity; its molecular weight is 488.15 and the purity was over 99% by HPLC analysis. Ethyl acetate and poloxamer were obtained from the National Pharmaceutical Group Chemical Reagent Co., Ltd., China; the drugs were prepared into solid dispersions with Poloxamer188 (Pluronics®) (drug:excipient = 1:9) and were orally administered by intragastric intubation by dissolving the solid dispersions in sterile water from days 7 to 21. The rats in the normal control and vehicle control groups were given excipient solution of the same concentration.

### Instruments

Toe volume meter (YLS-7C) was from Ji’nan Yi Yan Technology Development Co., Ltd (China). The electronic balance was from Mettler Toledo (U.S.A.). The centrifuge was from Eppendorf (Germany). The microplate reader was from Thermo (U.S.A.). The −80°C ultra-low temperature freezer was from Haier Bio-Medical (China). The electrophoresis apparatus was from Bio-Rad (U.S.A.). The transfer box was from Thermo Fisher Scientific (U.S.A.).

### Arthritis scoring

Arthritis severity was scored under blinded conditions, with each paw assigned a clinical score as follows: 0 = normal; 1 = mild erythema and swelling were limited to ankle and toe; 2 = mild erythema and swelling ankle extension; 3 = severe swelling was extended from the ankle to the metatarsal joint and erythema; and 4 = deformity or ankylosis [20].

### Edema measurement

Arthritis edema was measured by a plethysmograph measurement of the plantar volume (Friendship Technology Development Co., Ltd, Jinan, China). Primary and lateral (contralateral) secondary reactions after the injection of FCA in rats were recorded every 3 days. Each foot was measured three times, and the average was recorded [20].

### Enzyme-linked immunosorbent assay to measure levels of TNF-α, IL-6, IL-1β and IL-10 in synovial fluid

After treatment, the rats were anesthetized. Under anesthesia,the rats were injected with 0.5 ml saline into the joint cavity; 0.5 ml synovial fluid was drawn and centrifuged at 12000 rpm for 10 min to collect the supernatant. For the determination of TNF-α (Cat# EK0526, 15.6–1000 pg.ml^−1^), IL-6 (Cat# EK0412, 62.5–4000 pg.ml^−1^), IL-1β (Cat# EK0393, 31.2–2000 pg.ml^−1^) and IL-10 (Cat# EK0418, 31.2–2000 pg.ml^−1^) concentration in plasma, an enzyme-linked immunosorbent assay (ELISA) kit from Wuhan Boshide Biological Engineering Co. Ltd was used. Assay procedures were as described in the product manual, using the internal standard calibration curve to calculate the results [21]. Optical density (OD) of the samples was measured at a wavelength of 405 nm using a microplate reader (Thermo, U.S.A.).

### Histological analysis

The left hind paw joints were fixed in 10% formalin and were decalcified in 10% ethylene diamine tetra acetic acid (EDTA) for 6 weeks at 4°C [22]. Paraffin sections were stained with Hematoxylin and Eosin (HE). Sagittal sections (5 μm) were analyzed using an inverted microscope equipped with digital cameras (Leica DM2500, Leica Microsystems, Wetzlar, Germany) for evaluation of cell infiltration, pannus formation and cartilage damage [23]. Six sections of each group were randomly selected under a microscope to observe the cell morphology. Sections were evaluated histopathologically and scored for cartilage erosion, according to published criteria. Briefly, for cartilage erosion score 0: no destruction; score 1: minimal erosion limited to single spots; score 2: slight to moderate erosion in a limited area; score 3: more extended erosions and score 4: general destruction [24].

### Western blot to detect MAPK protein expression in articular soft tissues

On the 21st day, at the end of the experimental period, the animals were killed by cervical dislocation. After the cartilage tissue was ground with liquid nitrogen, RIPA lysis buffer containing 1% PMSF (Beyotime, Tianjin, China) was added to extract the total protein. Proteins were separated on 10% polyacrylamide gels electrophoresis (1.5-mm-thick) using 4% sodium dodecyl sulfate (SDS) gels according to the standard procedure of Laemmli [25]. Fifty micrograms of protein were loaded on to SDS/PAGE gels and then transferred to PVDF membranes. Membranes were blocked with 5% milk in Tris-buffered saline/Tween 20 (TBS-T) for 3 h and were incubated overnight (4°C) with antibodies (1:1000) against p-IKKα/β (Ser^176/180^, 85/87 kDa, Cell Signaling Technology, Cat# 2697), Ikkβ (87 kDa, Cell Signaling Technology, Cat# 8943), p-IκBα (Ser^32^, 40 kDa, Cell Signaling Technology, Cat# 5209), p-NF-κB p65 (Ser^536^, 65 kDa, Cell Signaling Technology, Cat#3033), NF-κB p65 (65 kDa, Cell Signaling Technology, Cat# 8242), JNK (46/54 kDa, Cell Signaling Technology, Cat# 9252), p-JNK (Thr^183^/Tyr^185^, 46/54 kDa, Cell Signaling Technology, Cat# 9251), p38MAPK (43 kDa, Cell Signaling Technology, Cat# 9212), p-p38MAPK (Thr^180^/Tyr^182^, 43 kDa, Cell Signaling Technology, Cat# 9216), ERK1/2 (42/44 kDa, Cell Signaling Technology, Cat# 4696), p-ERK1/2 (Thr^202^/Tyr^204^, 42/44 kDa, Cell Signaling Technology, Cat# 4370) or β-Actin (45 kDa, Cell Signaling Technology, Cat# 4970) were incubated with the membranes overnight at 4°C on a shaker. Membranes were washed with phosphate-buffered saline (PBS) and incubated the secondary antibody horseradish peroxidase (HRP)–conjugated goat anti-mouse (1:5000) for 2 h at room temperature [26]. All experiments were performed in triplicate and the results were reproducible. The membranes were scanned on an Image Station 4000MM (Carestream Health, Inc, U.S.A.). Images were analyzed with ImageJ software.

### Statistical analysis

The statistical significance was analyzed by Tukey’s test model of ANOVA (SPSS-20.0 software; IBM Corp., Armonk, NY, U.S.A.). *P*<0.05 was considered statistically significant. The results were expressed as means ± SE.

## Results

### Effect of FM0807 on symptoms in AIA rats

To investigate the pharmacological effects of FM0807 on RA development, an AIA model in SD rats was established. The rats were induced by injecting with FCA. They developed acute inflammation including redness, swelling, heat and joint dysfunction in the injected paw on day 2–3 after FCA injection. Symptoms were reduced after day 3–7. With deterioration in symptoms, multiple joint inflammations occurred gradually. Some rats developed erythema and inflammatory nodules in the ears and tail at various stages of disease, accompanied by extra-articular symptoms including hair loss, nasal congestion and bleeding.

**Figure 2:**
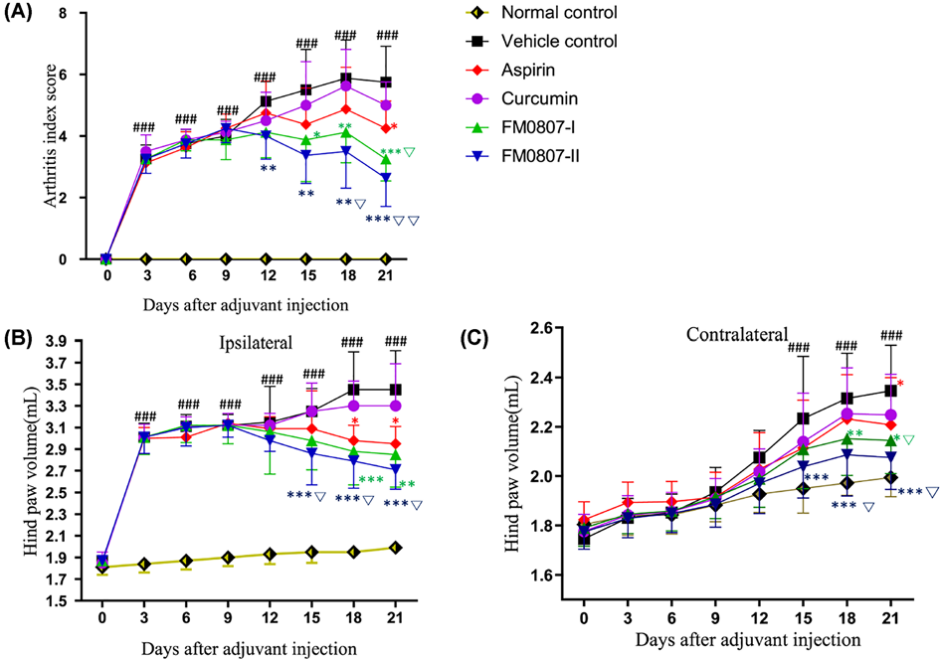
Effect of FM0807 on symptoms in AIA rats SD rats were immunized by a single intradermal injection of 0.1 ml FCA into the left hind paw to establish the AIA model. Immunized rats received FM0807 (0.1 or 0.2 mmol.kg^−1^ per day), curcumin (0.1 mmol.kg^−1^ per day) or vehicle by gastric gavage daily from day 7 to 21. A group of immunized rats receiving aspirin (0.1 mmol.kg^−1^ per day) during the same period served as positive controls. Clinical evaluation was performed prior to immunization (baseline) and on alternate days after the initiation of FM0807/Cur/Aspirin treatment (post-dosing) up to 21 days through standardized scoring of arthritis (**A**), measurement of edema (**B,C**). The results are shown as mean ± SD; *n*=8 per group. *P<0.05, **P<0.01, ***P<0.001 *vs.* the vehicle control; ^###^*P*<0.001 *vs.* the normal control; ^∇^*P*<0.05, ^∇∇^*P*<0.01 *vs.* the aspirin group. Data were analyzed using one-way ANOVA, followed by *t* test.

No significant differences were found in hind paw volume and arthritis index (AI) score in all groups prior to the immunization. The injected hind paws showed acute inflammatory swelling over a 24-h period in the foot injected with adjuvant. The ipsilateral hind paw volume increased over the next 12 days (*P*<0.01 Figure 2A), followed by subsequent chronic polyarthritis; concurrently, inflammatory edema in the contralateral hind paws also become significant (*P*<0.05 on days 15–21, Figure 2C). The mean AI score increased slightly during the first 9 days and subsequently rose further, up to 21 days after immunization (Figure 2A). Administration of FM0807 did not lead to significant inhibition of the development of joint swelling induced by the adjuvant in the acute phase; the ipsilateral and contralateral hind paw volumes were not significantly different relative to those of rats treated with the vehicle. However, 15 days after immunization (8 days after the termination of the treatment), the mean AI scores for all FM0807 treated groups (0.1 and 0.2 mmol.kg^−1^) were significantly lower (*P*<0.05, Figure 2A) than those of the vehicle group. FM0807 inhibited the ipsilateral hind paw in a dose-dependent manner (*P*<0.01 *vs* vehicle control for 0.1 mmol.kg^−1^ at 18 and 21 days; *P*<0.01 *vs*. vehicle control for 0.2 mmol.kg^−1^at 15, 18 and 21 days, Figure 2B). FM0807 also inhibited the contralateral hind paw (*P*<0.05 *vs*. vehicle control for 0.1 and 0.2 mmol.kg^−1^ at 18 and 21 days, Figure 2C). On the other hand, aspirin significantly restricted the development of AIA, as indicated by hind paw volume and AI score, but its effect was weaker than that of FM0807-I and FM0807-II.

### Effects of FM0807 on cytokine levels in AIA rats

**Figure 3:**
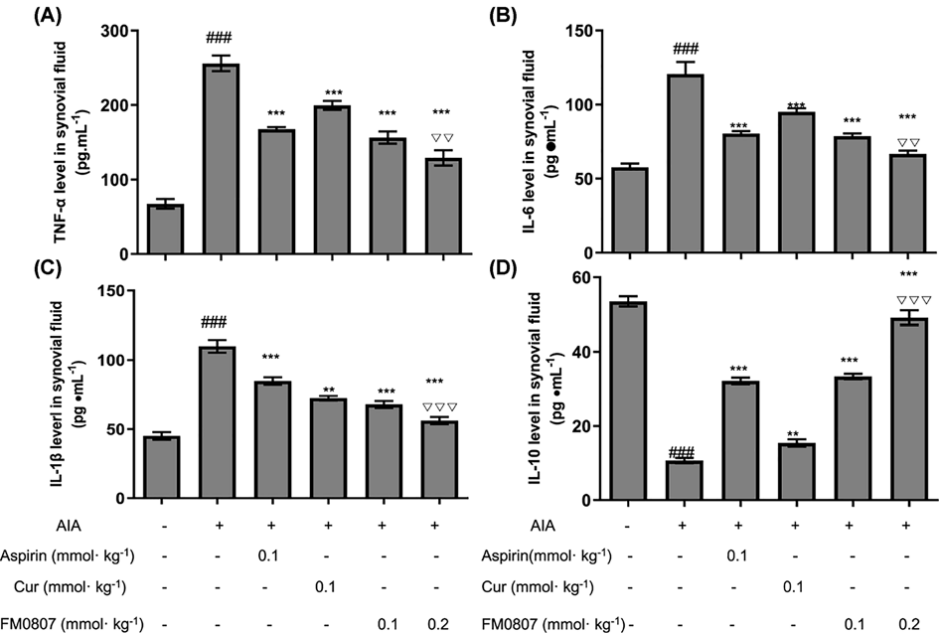
Effects of FM0807 on Cytokine Levels in AIA rats The animals were treated as described in Figure 2. Synovial fluid samples collected after completion of treatment were assayed for TNF-α (**A**), IL-6 (**B**), IL-1β (**C**), and IL-10 (**D**) using a commercially available ELISA kit. Data are presented as mean ± SD; *n*=8 per group. **P<0.01, ***P<0.001 *vs.* the vehicle control; ^###^*P*<0.001 *vs.* the normal control; ^∇∇^*P*<0.01, ^∇∇∇^*P*<0.001 *vs.* the aspirin group. Data were analyzed using one-way ANOVA, followed by *t* test.

Cytokine production is an important part of the inflammatory response. We measured the effect of FM0807, aspirin and curcumin on secretion of various pro-inflammatory cytokines, including TNF-α, IL-6, IL-1β and IL-10 in rats with AIA. The levels of TNF-α, IL-6, IL-1β and IL-10 in synovial fluid were measured by ELISA (Figure 3). Compared with the normal control group, the levels of pro-inflammatory cytokines TNF-α (Figure 3A), IL-6 (Figure 3B) and IL-1β (Figure 3C) were significantly higher in AIA vehicle control groups, while levels of the anti-inflammatory cytokine IL-10 (Figure 3D) were lower. Compared with AIA vehicle control groups, the expression levels of TNF-α, IL-6 and IL-1β deposition were lower in FM0807-I (*P*<0.01), FM0807-II (*P*<0.001) and aspirin (*P*<0.01) groups. IL-10 level was elevated in FM0807-I (*P*<0.001), FM0807-II (*P*<0.001) and aspirin (*P*<0.01) groups. These results suggest that FM0807 might exert its anti-arthritic agent through inhibition of expression of inflammatory factors.

### Effects of FM0807 on joint destruction and inflammation in AIA rats

**Figure 4:**
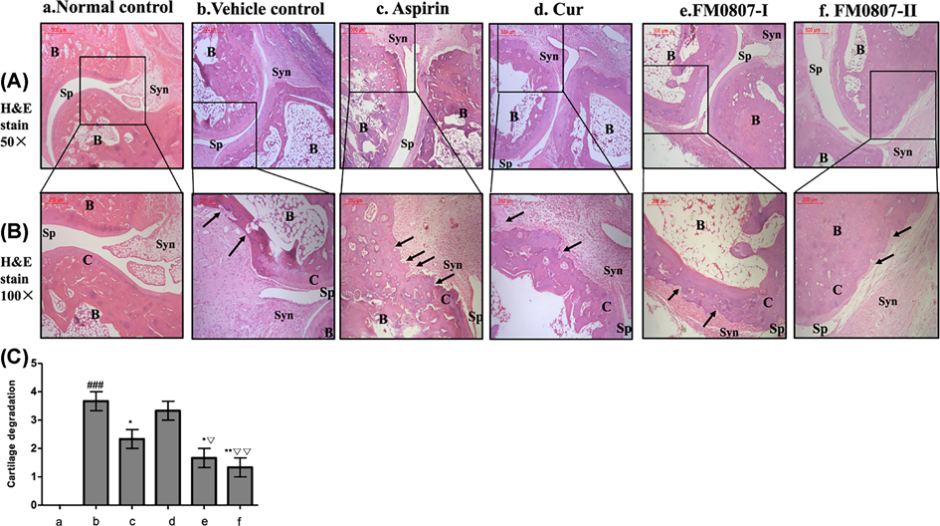
Effects of FM0807 on joint destruction and inflammation in AIA rats The animals were treated as described in Figure 2. The hind limbs of rats from all groups were examined by HE staining of the ankle joint upon completion of treatment and all behavioral tests. B, bone; C, cartilage; Sp, joint space; Syn, synovial tissue, black arrow, pannus. (**A**) Original magnification: 50×. (**B**) Original magnification: 100×. (**a**) Normal control showed the normal joint structure; (**b**) there was serious cartilage degradation in vehicle control rats; (**c**) Aspirin administration reduced the cartilage degradation; (**d**) Curcumin treatment showed no significant inhibition upon but, (**e**) FM0807-I (0.1 mmol.l^−1^)-treated group showed loss of pannus-formation in the cartilage and (**f**) FM0807-II (0.2 mmol.l^−1^)-treated group ceased the cartilage degradation. (c) The graph denotes the mean score of cartilage degradation based on the observation and assessed on a semi-quantitative four-point scale (score 0: no destruction; score 1: minimal erosion limited to single spots; score 2: slight to moderate erosion in a limited area; score 3: more extended erosions and score 4: general destruction). Results are presented as mean ± SD. *P<0.05, **P<0.01 *vs.* the vehicle control; ^###^*P*<0.001 *vs.* the normal control; ^∇^*P*<0.05, ^∇∇^*P*<0.01 *vs.* the aspirin group. Data were analyzed using one-way ANOVA, followed by *t* test.

Destruction of periarticular bone and articular cartilage are dominant features of pannus formation and joint swelling. We used HE staining to evaluate the degree of AIA joint lesions (Figure 4a–f). The histological evaluation using semi-quantitative four-point scale is shown in Figure 4C. The joint surface of normal control (Figure 4a) was smooth, the arrangement of chondrocytes was regular and the matrix was homogeneous. Histological examination of vehicle control revealed a large number of infiltrated inflammatory cells in the synovial space, along with granulation tissue. The articular cartilage was damaged. In particular, there was severe pannus formation in the synovial membrane, cartilage, and subchondral bone junction areas (*P*<0.001 *vs.* normal control, Figure 4b). By contrast, there was a significantly lower degree of proliferation of synovial cells as well as less inflammatory cell infiltration in all FM0807-treated animals. Furthermore, pannus was not apparent and matrix distribution was uniform in a dose-dependent manner (*P*<0.05 *vs* vehicle control for 0.1 mmol.kg^−1^, Figure 4e,C; *P*<0.01 *vs* vehicle control for 0.2 mmol.kg^−1^, Figure 4f,C). The aspirin group also had less histological damage of joints. The infiltration of inflammatory cells in the synovial membrane was significantly lower than in the vehicle control (*P*<0.05, Figure 4c,C), but there was also substantial damage to the cartilage and subchondral bone or pannus formation. Taken together, the histological data suggested that administration of FM0807 ameliorated the degeneration of bone and cartilage in joints of AIA rats.

### FM0807 inhibition of the MAPK and NF-κB signal pathways in AIA rats

**Figure 5:**
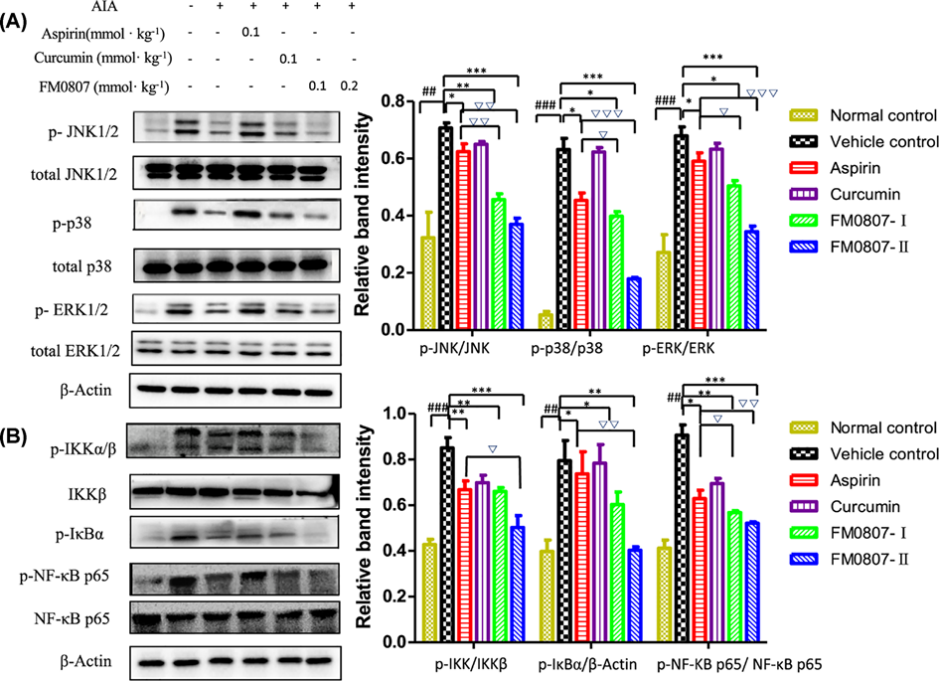
Effects of FM0807 on MAPK and NF-κB signal pathways in AIA rats The animals were treated as described in Figure 2. (**A**) FM0807 inhibited the phosphorylation of JNK, ERK and p38MAPK in a dose-dependent manner. (**B**) FM0807 inhibited the phosphorylation of IKK, IκB and NF-κB p65 in rats with AIA. The values are expressed as mean ± SD. *P<0.05, **P<0.01, ***P<0.001 *vs.* the vehicle control; ^##^*P*<0.01, ^###^*P*<0.001 *vs.* the normal control;^ ∇^*P*<0.05, ^∇∇^*P*<0.01, ^∇∇∇^*P*<0.001 *vs.* the aspirin group. Data were analyzed using one-way ANOVA, followed by *t* test.

To investigate whether the inhibition effect of FM0807 on AIA was related to the modulation of the MAPK and NF-κB signal pathways, we measured total JNK1/2, p38, ERK1/2, NF-κB p65, Ikkβ and phosphorylated-JNK1/2, p38, ERK1/2, NF-κB p65, IKKα/β and IκBα protein levels by Western blot analysis. The relative protein levels were quantified densitometrically using ImageJ software, and β-actin was used as a housekeeping protein. Typical examples of protein expressions from various groups are shown in Figure 5. As shown in Figure 5A, phosphorylation of JNK1/2, p38 MAPK and ERK1/2 was elevated in the AIA model (*P*<0.01 *vs* normal control). FM0807 treatment suppressed phosphorylation of JNK1/2 (*P*<0.01 for 0.1 mmol.kg^−1^, *P*<0.001 for 0.2 mmol.kg^−1^), p38MAPK (*P*<0.05 for 0.1 mmol.kg^−1^, *P*<0.001 for 0.2 mmol.kg^−1^) and ERK1/2 (*P*<0.05 for 0.1 mmol.kg^−1^, *P*<0.001 for 0.2 mmol.kg^−1^) compared with the vehicle control group. Total levels of ERK1/2, JNK1/2 and p38 were not changed following FM0807 treatment (*P*>0.05 *vs* vehicle control). These results suggested that the inhibitory effect of FM0807 on AIA rats was mediated by the MAPK pathway. As shown in Figure 5B, the inhibitor of NF-κB kinase (IKK)/IκB/NF-κBp65 signaling pathway may be activated in AIA rats (*P*<0.01 *vs* normal control). However, FM0807 inhibited the expression of p-IKKα/β, p-IκBα and p-NF-κB p65 (all *P*<0.01 for 0.1 mmo.kg^−1^, *P*<0.001 for 0.2 mmol.kg^−1^). The downstream effectors of the JNK, p38MAPK and ERK MAPK signal pathways are well-known [27].

## Discussion

RA is an autoimmune disease characterized by abnormal proliferation of synovial membrane cells and inflammatory cells infiltration, leading to whole joint synovitis and pannus formation [28]. In advanced stages, RA results in synovial thickening, invasive pannus formation, erosion of cartilage and bone and ultimately joint destruction. AIA was chosen as an experimental model for RA because it shares a number of pathological, histological and immunological features with human RA. AIA was induced by injecting with FCA containing heat-killed *Mycobacterium tuberculosis* (MT) via a mechanism involving heat shock proteins (HSPs) [29]. The joint inflammation and pathological changes are those seen in humans, as has been described extensively in the literature [30].

It is well-known that oral administration is the most widely used and most readily accepted route for patients, owing to its high patient compliance and flexibility. However, the oral bioavailability of curcumin is very low, greatly limiting its clinical use. Therefore, a new dosage form that can improve curcumin’s bioavailability and stability is highly desirable [31]. Curcumin is an effective anti-arthritic agent, and the present nanoemulsions appear to be promising systems, allowing RA therapy with curcumin to be converted from IV into oral administration [12]. In the current study, solid dispersion of FM0807 and its oral bioavailability were increased compared with that of curcumin, resulting in better anti-arthritic effects.

Cytokines are important mediators of inflammation and joint damage of RA. TNF-α, IL-6, IL-1β and IL-10 are closely related to pathological processes in RA [32]. High levels of TNF-α, IL-6, IL-1β and low levels of IL-10 are seen in RA patients, produced mainly by synovial cells and cartilage [33]. These cytokines activate inflammatory cells, stimulate infiltration of neutrophils and plasma cells and promote inflammatory granuloma formation. Furthermore, bone and cartilage destruction caused by these cytokines promote proliferation of synovial cells, fibroblasts and blood vessels with formation of typical pannus. We demonstrated substantial pannus formation and infiltration of inflammatory cells in the vehicle group. ELISA revealed that FM0807 reduced expression of IL-1β, IL-6, TNFα, and up-regulated expression of IL-10, to varying degrees with concentration dependence.

NF-κB, one of the major transcription factors implicated in joint inflammation [34], is essential for the production of cytokines and proteases by RA. In typical NF-κB signaling, after activating the upstream IKK, it combines with IκB and phosphorylates it. Then, the p65 subunit of NF-κB is released into the nucleus, where it modulates target genes. Then, the subunit is phosphorylated and transferred into the nucleus [35,36]. IKK/IκB/NF-κB signaling can make a difference in adjusting inflammation [37]; therefore, we determined whether FM0807 had an inhibitory effect on inflammation. We found that FM0807 reduced expression levels of p-IKK, p-IκB and p-NF-κB in the AIA model.

Furthermore, we determined that FM0807 influenced the activation of MAPK. The phosphorylation of all three kinases (ERK, JNK and p38MAPK) in the MAPK pathway was up-regulated by inflammation. The p38MAPK pathways are major targets of drug discovery in arthritis. The p38 cascade regulates pro-inflammatory genes post-transcriptionally; however, it remains a controversial target due to modest efficacy and severe toxicity in clinical trials [38]. C-Jun N-terminal kinase (JNK), also known as the stress-activated protein kinase (SAPK), was discovered in 1990 as an MAPK, a member of the MAPK family. The JNK signaling pathway may be activated by many factors, including cytokines involving TNF-α and stress involving ionizing radiation, osmotic pressure, heat shock and oxidative damage [39]. All these pathways participate in cell proliferation and differentiation, cell morphology maintenance, cytoskeleton construction, apoptosis and cell proliferation. In recent studies, Ma et al. [40] found that TNF-like protein 1A (TL1A) promoted formation of IL-6 in fibroblasts of RA patients, probably playing a role through activation of the JNK signaling pathway. In the present study, we showed that FM0807 inhibited expression of JNK in the articular soft tissues of AIA rats to varying degrees. The inhibitory effects on JNK were substantial in the FM0807 (0.1 or 0.2 mmol.kg^−1^) group, consistent with TNF-α and other inflammatory factors. The ERK pathway is a signaling cascade ubiquitously expressed in eukaryotic cells. It is thought to be primarily regulated by cell growth and proliferation, and it is also believed to mediate inflammatory responses [41]. ERK is continuously activated in RA synovium. ERK is closely associated with the inflammatory responses of RA, FLS (fibroblast-like synoviocytes), and bone destruction in arthritic joints [42]. Blockage of ERK with FR180204 (a selective ERK inhibitor) reduced the clinical severity of collagen-induced arthritis in mice, suggesting that ERK signal might be a therapeutic target for RA [43]. In the present study, we observed increased phosphorylation of MAPKs in the AIA group. FM0807 treatment inhibited phosphorylation of ERK, p38 and JNK, suggesting a role for MAPK in the mechanism through which FM0807 may have anti-arthritic activity.

In summary, our results suggested that the curcumin derivative FM0807 reduced the number of pro-inflammatory factors and reduced the inflammatory reaction in AIA rats, mediated by activation of the NF-κB and MAPK signaling pathway. These discoveries may offer new insights into the anti-inflammatory activity of FM0807, and may probably be serviceable as a mechanism for restraining inflammation. It may introduce a new modality for treating RA.

## Abbreviations

AI: arthritis index
AIA: adjuvant-induced arthritis
DMARD: disease-modifying anti-rheumatic drug
ELISA: enzyme-linked immunosorbent assay
ERK: extracellular-signal-regulated kinase
FCA: Freund’s complete adjuvant
FM0807: curcumin salicylate monoester, 2-hydroxy-, 4-[(1E,6E)-7-(4-hydroxy-3-methoxyphenyl)-3,5-dioxo-1,6-heptadien-1-yl]-2-methoxyphenyl ester
HE: Hematoxylin and Eosin
IκB: inhibitor of NF-κB
IKK: inhibitor of NF-κB kinase
IL: interleukin
JNK: c-Jun-N-terminal kinase
MAPK: mitogen-activated protein kinase
MTX: methotrexate
NF-κB: nuclear factor κB
RA: rheumatoid arthritis
SD: Sprague–Dawley
SDS: sodium dodecyl sulfate
SE: Standard Error
SPF: Specific pathogen Free
TNF-α: tumor necrosis factor-α
